# Prototyping the Automated Food Imaging and Nutrient Intake Tracking System: Modified Participatory Iterative Design Sprint

**DOI:** 10.2196/13017

**Published:** 2019-05-09

**Authors:** Kaylen J Pfisterer, Jennifer Boger, Alexander Wong

**Affiliations:** 1 Department of Systems Design Engineering University of Waterloo Waterloo, ON Canada; 2 Schlegel-UW Research Institute for Aging Waterloo, ON Canada

**Keywords:** participatory iterative design, usability assessment, perceived workload, feasibility assessment, application-driven research, systematic prototyping, nutritional support, long-term care

## Abstract

**Background:**

A total of 45% of older adults living in long-term care (LTC) have some form of malnutrition. Several methods of tracking food and fluid intake exist, but they are limited in terms of their accuracy and ease of application. An easy-to-use, objective, accurate, and comprehensive food intake system designed with LTC in mind may provide additional insights regarding nutritional support systems and nutritional interventions.

**Objective:**

The aim of this study was to conduct a multistage participatory iterative design sprint of a Goldilocks quality horizontal prototype for the Automated Food Imaging and Nutrient Intake Tracking (AFINI-T) system. Specific design objectives included the following: (1) identify practice-relevant problems and solutions through user-centered participatory design, (2) mitigate feasibility-related barriers to uptake, and (3) employ user-centered technology development.

**Methods:**

A 6-stage iterative participatory design sprint was developed and executed. A total of 38 participants and advisors representing 15 distinct roles (eg, personal support worker, nurse, and dietitian) were engaged in the design sprint. Subjective workload (Raw Task Load Index), subjective usability scales, and a modified Ravden checklist were used to assess project advisors’ perceptions of the AFINI-T system prototype compared with the current method of food and fluid intake charting.

**Results:**

The top priorities for this system were identified as the following: ease of use, high accuracy, system reliability, ease of maintenance, and requirement of integrating with the current PointClickCare system. Data from project advisors informed design decisions leading to a Goldilocks quality horizontal prototype of the AFINI-T system. Compared with the current food and fluid intake charting system, AFINI-T was perceived to have the following: less time demands (*t*_10.8_=4.89; *P*<.001), less effort (*t*_13.5_=5.55; *P*<.001), and less frustration (*t*_13.0_=3.80; *P=*.002). Usability ratings of the AFINI-T prototype were high, with a subjective usability score mean of 89.2 and the highest ratings on a modified Ravden usability checklist of “very satisfactory” for 7 out of 8 sections.

**Conclusions:**

The AFINI-T concept system appears to have good practice relevance as a tool for an intelligent food and fluid intake tracking system in LTC. The AFINI-T concept system may provide improvement over the current system, and advisors are keen to try the AFINI-T system. This research gives tangible examples of how the sprint method can be adapted and applied to the development of novel needs-based application-driven technology.

## Introduction

### Background

The link between poor nutritional status and disease is well established; malnutrition is associated with decreased quality of life, increased hospital stays, pressure ulcers, morbidity, and mortality [1-3]. Furthermore, malnutrition-related costs the health care system US $10 billion per year in each the United States and United Kingdom [4,5]. Older adults are at increased risk of nutritional deficiency because of physical and physiological changes (eg, reduced lean muscle, less efficient gastrointestinal tracts, and changes in sensory ability such as smell or taste), in addition to having a higher degree of comorbidity [6]. Older adults living in long-term care (LTC) are particularly vulnerable; in Canada, 97% of older adults require assistance with activities of daily living (including eating assistance), 90% of the population is living with memory impairment, 61% of the population is on 10 or more medications, and 49% of the population is living with depression [7]; these demographics are similar in the United States [8]. On the basis of a recent Canadian study, approximately 44% of the LTC population is malnourished [9], which is consistent with a systematic review of global research (37 studies, 17 countries; malnutrition prevalence: 19% to 42%) [10]. Best practice metrics for ongoing nutritional assessment include monitoring unintentional weight loss, usual low intake of food, or other quality indicators to prioritize referrals and monitor effectiveness of nutritional support systems [11]. However, although inadequate intake is manageable [12], present guidelines for a nutritional intervention stipulate a resident must consume less than 75% of a meal most of the time [13-15]. Half of these residents who would benefit from an intervention are missed [14,15] because of difficulties assessing and charting food intake. Thus, monitoring nutritional status in LTC is crucial but difficult to do so effectively.

In LTC, nursing assistants or personal support workers (PSWs) chart food and fluid intake of residents using either a paper-based or an electronic form to capture intake across a meal at 25% incremental proportions of intake. The accuracy of these methods is known to be poor, with incorrect estimates over 50% of the time [16]. One contributing factor is time constraints in the LTC environment, and it is further confounded by frequent retrospective charting, which increases the probability of reporting errors [13]. Although accuracy is important to ensure appropriate referrals of residents to a registered dietitian (RD) [14], the current method fails to differentiate among aspects of a meal; equal consumption across a plate is assumed. To address this, Andrews and Castellanos developed a food-type specific tool; however, consumption was still underestimated 25% of the time [13]. The challenge remains that comparisons either require time-consuming methods or need to be completed by highly qualified personnel [14].

Technological innovations may provide a solution to remove subjectivity, enhance reproducibility, and inform higher levels of detail. There has been some progress in automatic food intake tracking systems. For example, several devices have been proposed for an individual to track and manage weight loss by recording intake using a mobile device [17-20]. Although this on-the-go approach could potentially be modified for appropriate use in LTC settings, in its current state, it is tailored for a different purpose, relies on self-monitoring, and does not adhere to related best practices for food and fluid intake. In addition, they require a series of images from multiple perspectives [17] or depend on reference objects to infer scale (ie, fiducial marker) [19]. In a time-constrained environment such as LTC and hospital settings, these requirements make these approaches infeasible. Consistent with this apparent gap, a 2016 review by Pouladzadeh et al [20] summarizes both traditional and newer (smartphone vision-based) methods for calorie intake tracking in the context of weight loss and weight maintenance. They conclude that several challenges remain, including the following: the explicit need for user acceptance studies of nutritional monitoring technology, consideration of more complex meal scenarios, and computational requirement consideration [20]. Within the LTC context, the closest technological solution was a comparison to estimate food waste of regular- and modified-texture diets either with the visual estimation method or by using digital photographs afterward [21].

### Objectives and Goals

The above highlights the need for an easy-to-use, accurate, and comprehensive food intake system designed with the LTC context in mind. The goal of this research was to collaborate with representative end users to design a novel prototype system for Automated Food Imaging and Nutrient Intake Tracking (AFINI-T). End users in this context were team members working in LTC, involved in monitoring resident food intake (eg, PSWs and RDs). We developed a Goldilocks quality horizontal prototype by accomplishing the following objectives: (Objective 1) identify practice-relevant problems through user-centered participatory design, (Objective 2) remove feasibility-related barriers to uptake, and (Objective 3) facilitate confidence in design decisions for user-centered technology development. Our guiding principle was to accelerate research to uptake of novel technological solutions through practice-informed research. Each of the 3 objectives outlined above had several goals as follows: (Goal A) understand workflow and the problem space including user perceptions of workload of the current system (Objective 1); (Goal B) conduct a needs assessment within the problem space (Objective 1); (Goal C) establish functional criteria for usability and feasibility, including user interface requirements (Objective 2); (Goal D) evaluate a user-driven, practice-relevant early-stage prototype to inform future directions, including user perceptions of workload, usability, and receptivity of the AFINI-T system prototype (Objective 3). The primary contribution of this study is the novel AFINI-T system design created through the participatory iterative design by (1) the identification of functionality requirements and design considerations, (2) the findings and insights from user testing, and (3) a demonstration of and reflection on the effectiveness of this participatory iterative design methodology with a multidisciplinary team of project advisors. The remainder of this paper is organized as follows: the combined Design Stages section presents the 6 stages used in the design process, along with related results and discussion for each stage, followed by a general discussion before closing with overreaching conclusions.

## Methods

### Overview

Our goal was to create a Goldilocks quality horizontal prototype. “Goldilocks quality” refers to having the “just right” amount of fidelity to elicit useful feedback from users without having to build an entirely functional prototype [22]. A horizontal prototype refers to a user interface–based design to allow user feedback on an early-stage conceptual walk-through of the process [23]. We implemented an iterative participatory iterative design process, modeled off the Google Sprint framework, to develop and evaluate this prototype for monitoring food and fluid intake in LTC [22,24]. The 6 stages of our process were the following:

1. *STAGE 1:* Design Ideation
2. *STAGE 2:* Reflect and Storyboard (*see Multimedia Appendix 1)*
3. *STAGE 3:* Storyboard Critiques (*see Multimedia Appendix 1)*
4. *STAGE 4:* Design of the Goldilocks Quality Horizontal Prototype
5. *STAGE 5:* Usability Assessment
6. *STAGE 6:* Final Validation

**Figure 1:**
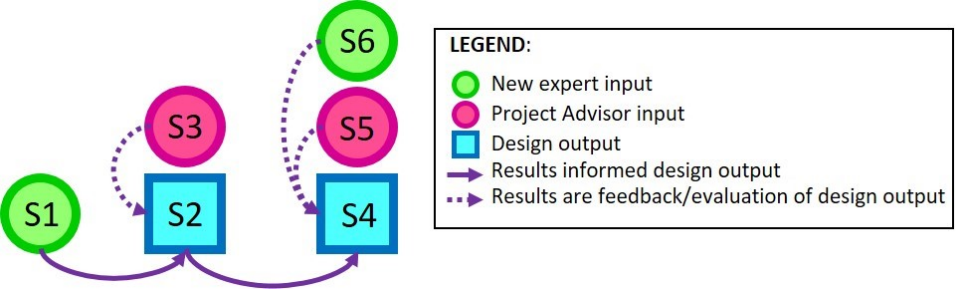
An overview of the 6 stages (eg, S1 means Stage 1), including information flow between stages. Solid arrows indicate results directly influencing design output (eg, S2’s story boards and S4’s Goldilocks prototype). Dashed arrows indicate feedback on a design stage. Feedback was collected from expert input (S1, S6 in green) and from ongoing project advisor engagement input (S3, S5 in pink).

The design process was guided by several conceptual frameworks: (1) conducting interdisciplinary research [25,26], (2) leveraging user-centered design and participatory design [27,28], (3) applying rapid prototyping methodology via a modified Sprint [22,23]; (4) applying best practices for user interface design [23,29-33]; and (5) evaluating usability [34,35] and perceived workload [36]. The flow of information through each stage is shown in Figure 1. For brevity, the methods (including collaborators, data captured, and analyses), results, and discussion for Stages 1 and 4 to 6 are presented below, within the context of each stage; details regarding Stage 2 and 3 can be found in Multimedia Appendix 1 [37-43].

### STAGE 1: Design Ideation – Methods

The purpose of Stage 1 was to engage with end users as collaborators to establish design directions. Specifically, we sought to understand the current workflow, evaluate priorities, understand the perceived workload of the current system, and identify potential project advisors. The output from this directly informed Reflect and Storyboard (Stage 2) and Usability Assessment (Stage 5).

#### Participants

Stage 1 comprised a 60-min workshop in which 3 activities were completed: *Activity 1: The “Ask the Experts” activity*; *Activity 2: Priority ranking survey completion*; and *Activity 3: “Vote with dots” exercise* to keep participants engaged and reflect on priorities. A total of 3 research assistants, plus the lead author, took notes during this discussion and transcribed several comments verbatim. Following the workshop, 3 informal open-ended interviews were conducted to further inform the problem space. The lead author took notes during these interviews; several comments were transcribed verbatim.

For the workshop, 21 participants representing 12 LTC and retirement homes were recruited through self-enrollment with the following roles: Administrative Assistant, Chef, Dining Lead (similar to a dining room manager), Director of Recreation, Dietary Aides, Neighborhood Coordinator, Recreation Assistant, Restorative Care, Senior Nurse Consultant, Directors and Assistant Directors of Food Services, Nurse, and PSWs. Activities were discussed with the Schlegel-UW Research Institute for Aging’s (RIA) Research Application Specialist for input on how to successfully conduct this exercise with front-line team members.

#### Tools

##### Activity 1: The “Ask the Experts” Activity

Workshop participants were asked about their experience with food and fluid intake. This aimed to build participants’ confidence in the value of their experiences while probing current workflow and problem space.

##### Activity 2: Priority Ranking Survey

Participants independently completed a survey to evaluate priorities and needs to limit bias. This survey asked about the current charting process (eg, when it is done, task completion time, and barriers and facilitators to task completion). For evaluating priorities, 5-point Likert scales were used to rate 16 statements’ importance from “Not Important” (ie, 0) to “Very important” (ie, 4) or “Not Applicable.” Perceived workload of the current system was retrospectively evaluated with the Raw Task Load Index (RTLX) [36,44] for its application simplicity and comparability to the NASA-TLX [44-47].

##### Activity 3: “Vote With Dots” Exercise

Modeled from the study by Knapp et al [22], participants transposed their individual Activity 2 responses into a group response by voting their preference using stickers on giant sticky notes to amalgamate opinions, keep participants engaged, and facilitate additional discussion.

#### Statistical Analysis

Given the nature and size of this pilot study, a preliminary thematic analysis was used for qualitative components (eg, discussions, comments, and verbal/written feedback) that was combined with descriptive statistics for quantitative information, including the average (µ), SD(_σ_), mode, and median scores [48]. For scales with 5 or more categories (eg, RTLX), µ(_σ_) is used; the mode was used for categorical data with fewer than 5 categories (eg, Ravden Checklist). A weighted average was used to analyze Likert survey questions, excluding “Not Applicable,” to yield a ranking of each statement.

### STAGE 4: Design of the Goldilocks Quality Horizontal Prototype – Methods

The purpose of Stage 4 was to create low-fidelity prototypes by incorporating the most promising solution concepts identified through the storyboard critiques in Stage 3. These prototypes were then used for pilot evaluation in Stage 5’s usability assessment.

#### Tools

Design decisions were informed by heuristics, as in Stage 2 [23,32,33], and feedback received from the storyboard critiques in Stage 3. The following heuristics were emphasized: universal usability was considered by testing the prototypes with different types of users (eg, academics and PSWs), providing informative feedback and error prevention, the output in this stage (Stage 4) was a Goldilocks quality horizontal prototype. This included interfaces for each of the 3 levels of primary users currently involved in residents’ food and fluid intake charting (ie, PSW, registered nursing team, and RD).

### STAGE 5: Usability Assessment – Methods

The goal of Stage 5 was to elucidate preliminary feasibility early on with end users through the evaluation of prototypes through pilot testing. The output from this stage informed how the prototypes could be improved for the development of a working system in the future.

Prototypes were evaluated by comparing perceptions of the AFINI-T prototype with the system currently in place with regard to usability and workload. Usability was assessed using the Subjective Usability Scale (SUS) [34] from the user perspective and a modified Ravden usability evaluation checklist [35] from technical experts’ perspectives; items pertaining to help, including all of section 9, were removed, as this was beyond the scope of the Goldilocks quality horizontal prototype.

#### Participants

A total of 4 project advisors from Stage 4 were tester participants (PSW, Dining Lead, Dietary Aide, and Nutrition Research Expert). By word of mouth, 2 new project advisors requested inclusion as observers for a total of 6 advisors. All testing was completed in person though one-on-one sessions. Testing sessions were audio-recorded and relevant quotes were transcribed verbatim. Testing began with an interview walk-through of the prototypes based on the script adapted from a study by Knapp et al [22] to ascertain usability and feasibility barriers. A novel, predefined strict set of tasks was completed by each advisor. The student investigator completed a checklist to capture the degree of success to which each task was completed (ie, success, required prompting, or failed).

#### Tools

The RTLX [36,44] was administered to enable comparison of perceived workload of the current method in place with the AFINI-T system prototype (Table 1). Usability was assessed with the SUS, which was selected over other usability questionnaires for its ease of use, minimal training requirements, and low application time [45,49]. The RTLX and SUS were also completed by the 2 observers (Director and Assistant Director of Food Services) based on their experience during the observation. These 2 project advisors had no previous experience or knowledge of this project.

For evaluating usability more formally, an adapted Ravden checklist was used by 2 technical experts with backgrounds in systems design engineering and limited exposure to the users’ perspectives. The Ravden checklist was selected for its low cost and ease of use to assess the interface with good interrater reliability and predictive validity [45,49] (Multimedia Appendix 3).

#### Statistical Analysis

A 2-tailed *t* test assuming unequal variances [50,51] was conducted to compare the current system and the AFINI-T system for users’ perceived workload for the RTLX. Quantitative data were analyzed using descriptive statistics, with highlights from qualitative data as described in Stage 1.

### STAGE 6: Final Validation – Methods

The goal of Stage 6 was to receive additional feedback from a group of RDs, directors, and assistant directors of food services to provide a fresh perspective to minimize bias.

#### Participants

The RDs, directors, and assistant directors of food services from across the Schlegel Villages were invited to participate in a webinar outlining the progress to date, along with tandem survey completion for assessing perceived usability and workload. A total of 13 people participated in the webinar (43% participation rate), which is consistent with the typical attendance of quarterly dietitian meetings at Schlegel Villages because of scheduling complexities.

## Results

### STAGE 1: Design Ideation – Results

Results from Stage 1 pertained to Objective 1: address a practice-relevant problem through user-centered participatory design (Goals A and B) and Objective 2: remove feasibility-related barriers to uptake and are as follows (Goal C):

#### Goal A: Understand Workflow and Problem Space

PSWs, registered nursing team, and RDs are primary users who conduct charting of food and fluid intake on iPads. This charting is completed whenever primary users have time, which could be during meal service or retrospectively, consistent with the study by Andrews and Castellanos [13]. In a follow-up discussion with the organization-wide director of food services, who is responsible for policy, she indicated that conducting food intake in real time is mandated (as opposed to retrospectively), but from the workshop discussion, it is clear there is a gap between policy and practice. Although the workflow of AFINI-T is congruent with this mandate, a solution to support this mandate in practice may require policy modifications. For example, a person may need to be assigned to the sole task of tracking food and fluid intake during mealtime, which means he or she would be unavailable to provide assistance with residents’ care needs for the duration of the meal. Changing policy is outside the scope of the current AFINI-T project but having sensitivity to this issue provides helpful context and informs that this may be a potential barrier to uptake of the system in practice.

Regarding the current system, respondents appreciated the ability to track fluids, so they need not manually add, and the output has units (mL). Although the current system is dependable, substantial barriers and limitations were identified regarding the effectiveness and accuracy of the current system. A workshop participant shared:

What’s being collected for solid food isn’t useful. It’s so high level and minimal can’t make use of it. [We] can’t infer anything regarding health or category of at-risk. [We] look at last 7 days, see “they had 75% of a meal so they're eating well”, but it doesn't say anything. [We] don’t get a lot of info from the charts.

Insufficient time, data inaccuracy, unreliability, and nonstandardized measurements were identified as the largest barriers for task completion. In addition, the inability to differentiate among types of foods and lack of relation to original serving size lead to data interpretation difficulties. For example, some residents prefer half portions; if they eat half of their portion, this could be recorded as 50% (ie, half of the serving they received) or it could be input as 25% (ie, one-fourth relative to the full portion). There is no guarantee that the proportion is input accurately or consistently. These themes were apparent through 2 sources, the “Ask the Experts” as well as on the survey. For more detail regarding the current system’s retrospective analysis of perceived user workload, see the sections of Table 1 pertaining to the “Current” system.

**Table 1:** Comparing retrospective perceived users’ workload measures of current food/fluid intake system from Stage 1 to the Automated Food Imaging and Nutrient Intake Tracking prototype results from Stage 5.

Workload demand^a^ and system		Mean	Mode(s)	Minimum	Maximum	Responses, N	*t* test (df)	*P* value
**Mental demand**								
	Current	10.2	6	4	19	10	2.56 (13.8)	.023
	AFINI-T^b^	4.4	3	1	10	6	2.56 (13.8)	.023
**Physical demand**								
	Current	6.4	2	1	15	9	1.41 (12.5)	.183
	AFINI-T	3.5	1	1	6	6	1.41 (12.5)	.183
**Time demand**								
	Current	16.7	19	5	20	10	4.89 (10.8)	<.001
	AFINI-T	5.5	3	1	12	6	4.89 (10.8)	<.001
**Performance**								
	Current	15.2	18, 20	3	20	10	0.722 (13.7)	.722
	AFINI-T	16.8	20	11	20	6	0.722 (13.7)	.722
**Effort**								
	Current	13.2	6	6	20	10	5.55 (13.5)	<.001
	AFINI-T	3.7	3	1	7	6	5.55 (13.5)	<.001
**Frustration**								
	Current	11.5	15	1	20	10	3.80 (13.0)	.002
	AFINI-T	3	2	1	8	6	3.80 (13.0)	.002

^a^Values could take on a range from 0 to 20; 0 implies no workload and 20 implies highest imaginable workload except in the case of performance which is reverse coded.

^b^AFINI-T: Automated Food Imaging and Nutrient Intake Tracking.

#### Goal B: Conduct a Needs Assessment of Problem Space Including Priority Areas

Workshop participants were asked to rate need statements’ importance. The top 3 ranked priorities were tied among (1) “ease of use” and “accuracy” (µ=3.9, mode: “very important,” 15 out of 16 votes), (2) “reliability” and “maintenance” (µ=3.9, mode: “very important,” 14 out of 16 votes), and (3) “The system should work well with PointClickCare” (µ=3.8, mode: “very important,” 12 out of 16 votes).

The following 5 themes emerged as wishes for a novel system to extend beyond the current infrastructure: (1) being able to leverage weight of food as a ground truth instead of relying solely on subjective proportions, (2) having the ability to track trends over time, (3) being able to discriminate among types of food, (4) being able to include fluid intake as well to discriminate between types of fluids, and (5) operating the system in different modes to accommodate various use cases (ie, in the dining room vs for in-room service). One additional, complementary theme relevant to priorities, identified independently through 3 interviews, was the need to support prioritizing referrals that consider symptoms and risk flags’ severity. One project advisor articulated:

There is 1 Registered Dietitian for 300 residents. It’s impossible to track properly … People are often missed because nurses aren’t identifying properly… If charting were accurate, this would help with the referral process.

#### Goal C: Establish Functional Criteria for Usability and Feasibility

The current system mode time to complete the task defined the time completion target: 10 to 14 min, maximum, per neighborhood (ie, “ward”) comprising 16 residents. Of the 21 workshop attendees, 11 self-identified as being involved in charting resident food and fluid intake and were asked about the amount of time required to complete intake charting for each type of food, fluid, or snack. Survey responses are outlined in Table 2.

**Table 2:** Summary of length of time required to complete food and fluid intake charting for 1 neighborhood comprising 16 residents (Stage 1).

Charting type	Mode time (min)	Responses^a^, n/N (%)	Time range (min)
Food (meal)	10 to 14	3/9 (30)	<10 to 25+
Fluid	10 to 14	4/10 (40)	<10 to 25
Snack	<10	5/9 (64)	<10 to 19

^a^n is the number of responses with the mode rating out of N, the total number of responses.

### STAGE 4: Design of the Goldilocks Quality Horizontal Prototype – Results

**Figure 2:**
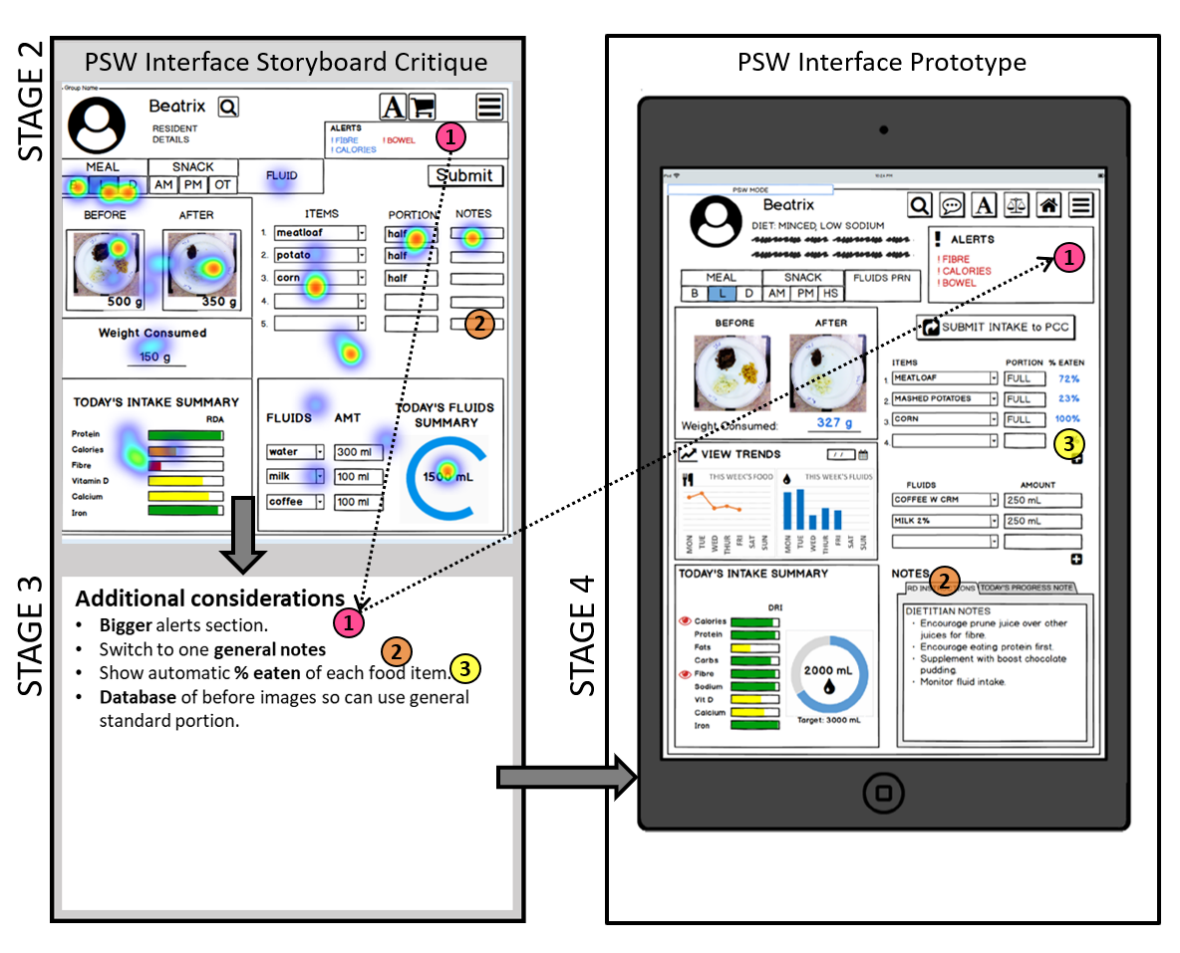
Stage 2 personal support worker user interface. Output from Stage 3 included a heat map on the most promising aspects (red indicates more votes, n=5) with qualitative feedback highlights for additional considerations. The right pane illustrates an example of the prototype interface. Numbers correspond to the flow of information and adapted feedback from Stage 2 through to 3 and 4 using the first example (#1 in pink) to further illustrate flow with the dashed arrow.

**Figure 3:**
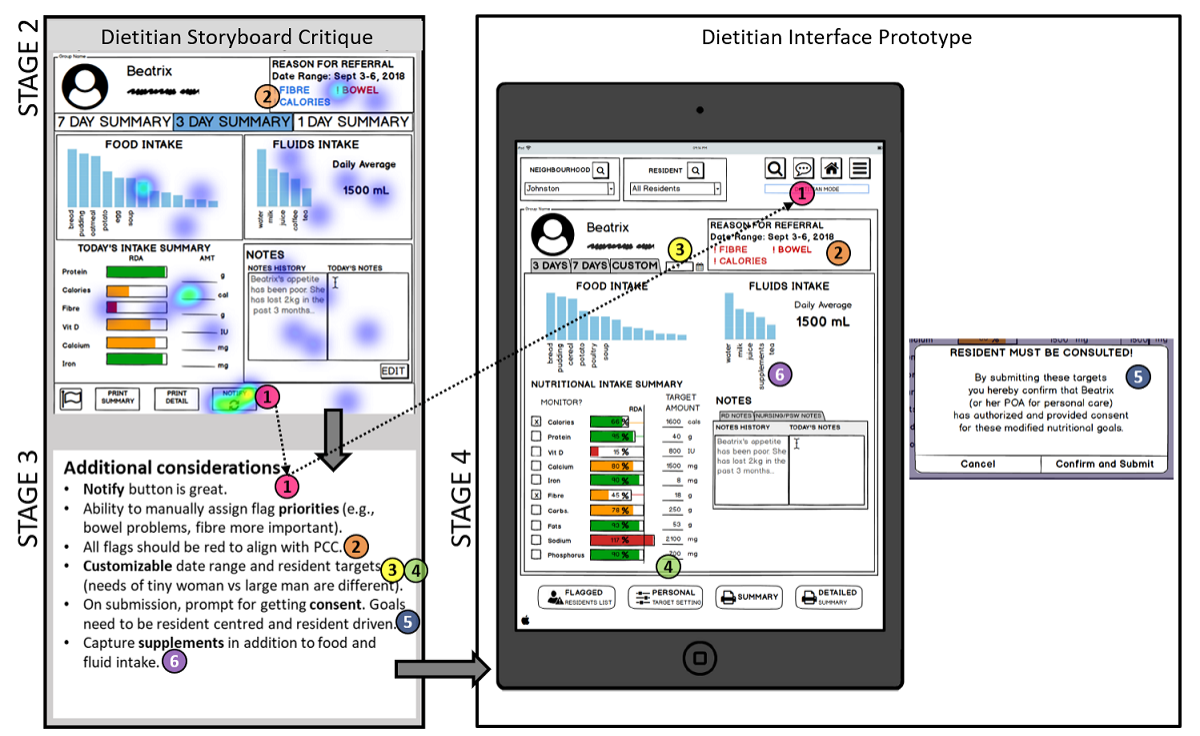
Stage 2 Registered Dietitian user interface. Output from Stage 3 included a heat map on the most promising aspects (red indicates more votes, n=5) with qualitative feedback highlights for additional considerations. The right pane illustrates an example of the prototype interface with a sample pop-out box. The numbers correspond to the flow of information and feedback from Stage 2 through to 3 and 4 using the first example (#1 in pink) to further illustrate flow with the dashed arrow.

Design heuristics were applied in the 4 ways, and sample output from this stage is illustrated in the right pane of Figures 2 and 3 with additional inspiration from commercially available online health care tools (Multimedia Appendix 2) First, related to universal usability, mapping was considered through matching the system with users’ language and familiar concepts in reality (eg, Figure 2 contains tab names for snacks, such as “AM,” “PM,” and “HS”, which refer to the morning, afternoon, and evening snacks, respectively) [23,32]. Second, informative feedback on a change of state was provided [23,33] when users attempted to submit or track an action; there is a pop-up banner at the bottom of the screen (not shown). Third, error prevention [23,32,33] was incorporated through limiting types of responses and providing feedback. For example, the PSW interface would prompt for a picture or a progress note before submission, with the ability to finish charting at a later point of the meal service. Fourth, efforts were made to reduce short-term memory load and enhance visibility/discoverability [23,32,33] by placing the workspace into panes, with all information accessible on 1 screen. Other features included making “smart” suggestions when selecting items or filling out portion sizes. For example, notes entered from the RD interface (not shown) would auto populate on the RD instructions tab in the PSW interface.

### STAGE 5: Usability Assessment – Results

Stage 5 results address Objective 3: Facilitate confidence in design decisions and empower user-centered technology development (Goal D).

#### Goal D: Evaluate a User-Driven, Practice-Relevant Prototype

Subjective usability was rated as “acceptable” with an average SUS score of 89.2, with the lowest and highest SUS scores of 72.5 and 97.5, respectively, translating to a B+ on the grade scale [52,53]. Mapping these scores onto the adjective ratings as described by Bangor et al, the majority of usability scores (5 out of 6) therefore fell between “excellent” and “best imaginable.” In line with these quantitative results, users commented that, “It’s quite intuitive, the key things were easily found,” “It’s a lot but it’s easy to learn and it’s colourful,” “I’m not technologically inclined, but most things I was able to do intuitively,” and “I think someone could use this if they were just thrown onto the floor with it.”

As highlighted in Table 1, performance was rated comparably, with an average score of 16.8 and 15.2 for the AFINI-T and current systems, respectively. In the case of mental demand, time demand, and effort and frustration, subjective workload ratings were significantly lower for the AFINI-T system than the current system (*P*<.05). These results suggest the AFINI-T system is perceived to require less effort and lower overall workload than the current system. This is consistent with comments from the participants including the following: “[This would take a] huge burden off me as a clinician. This is hugely better than paper… there are no guestimates… I don’t have to do work.” and “It makes life so much easier.”

For the AFINI-T system prototype in Stage 5, receptivity to the prototype was positive, with several areas identified for improvement. For example, the following was said regarding the general concept for the dietitian interface: “[It] would be good to personalize these specific needs and set it so the flags sent to nursing/PSW for these items based on what dietitian enters …This would save *a lot of time* especially if individualized.”; “Capturing [supplement intake] would enable dietitians to monitor intervention adherence … If it shows up that they never have it, then great feedback to change the intervention.”

A total of 2 technical experts completed a modified Ravden usability checklist evaluation with favorable ratings (Multimedia Appendix 3). Ratings across both raters for sections 1 to 8 were very satisfactory (7 out of 8 sections) or split among “satisfactory” and “very satisfactory” (1/8 sections) and mode for section 10 on system usability of “no problems.” Consistent with comments from user testing, the main suggested area for improvement was to increase customizability options (eg, sort resident list in multiple ways, allow more flexibility in the order of operations such as allow charting before a picture is taken).

### STAGE 6: Final Validation – Results

Receptivity of participants in Stage 6 was generally positive. The main reservation pertained to how the system would integrate with the current method and PointClickCare (corroborated in Stages 1, 5, and 6) and, more generally, the workflow. For example, 3 webinar participants’ direct messages were as follows: (1) “I love the idea of this system, we are concerned about workload, as well as if the systems (AFINI-T and PCC) talk to each other”; (2) “Would this be a separate system that would be linked to PCC?”; and (3) “I hope a PCC progress note is generated from any notes [a registered dietitian] adds.”

Finally, participants expressed reservations regarding the proposed AFNI-T system. One dietitian expressed concern about overemphasizing the importance of nutrition “in a population that should have the main focus of just making sure [residents] are enjoying the food we are serving.” There was also concern over how this will translate to Ontario Ministry of Health and Long-Term Care (MOHLTC) inspectors’ inspections and the perception that using a system like this will take more time. In addition, it was stated that there was no perceived value to having access to more detailed nutrient data in the LTC population as, to them, the largest issue contributing to malnutrition is the impact dementia has on the calories consumed. However, they did suggest that if there was an ability to screen for residents to focus on only those at greater risk for malnutrition that the AFINI-T system would be helpful while still meeting the MOHLTC standards, as only those at risk for malnutrition are mandated to track food and fluid intake. This provides an interesting complementary perspective and warrants further probing and discussion.

## Discussion

### Summary

The overall purpose of this study was to investigate the gap for user acceptance studies and work toward a feasible food and fluid intake tracking solution for use in LTC through a participatory iterative design process and the creation and evaluation of a Goldilocks quality horizontal prototype. Specific contributions of this study were the following: (1) identify practice-relevant problems and solutions through user-centered participatory design, (2) remove feasibility-related barriers to uptake, and (3) facilitate confidence in design decisions and empower user-centered technology development.

We applied a rapid prototyping methodology via a modified Sprint process [22,23]. For the AFINI-T prototype, the data collection and design part of our modified sprint took place over 6 weeks rather than the suggested 5 days. This was because of the infeasibility of having an entire team of project advisors dedicated full time based on volunteered time, in addition to project advisors’ regular full-time responsibilities. The discussion below is meant to elucidate several challenges in applying this framework in the academic research environment. In addition, we deepen our reflection on feedback received on the perception of the necessity of nutrient intake tracking in LTC with particular emphasis on this need within the dementia context.

### Challenges of Applying the SPRINT Framework in Academic Research

#### Potential Challenges Around Organizing Activities

We were fortunate to have had our proposed workshop (Stage 1) accepted by the RIA and the Schlegel Villages as part of their annual Innovation Summit. This enabled us to gain momentum and build rapport from the in-person meeting and enabled many perspectives across several homes (within the same organization) to guide the direction for this project. If this infrastructure were not in place, coordinating the initial workshop would have been more challenging but not impossible with the following modifications. Initial discussion could have taken place with key stakeholders at targeted meetings (eg, quarterly dietitian meeting, and monthly team meetings). This would have required more travel and more time at the outset. The authors were also fortunate to have experience conducting applied research in the LTC environment. For others who may be newer to this approach, we recommend arranging a multiple day observation or volunteer experience to learn what the work environment is like to authentically understand the nuances of the needs and environment. We believe one key factor is to identify a necessary but highly inefficient and unreliable process.

#### Addressing the Need to Connect From a Distance

Many of the SPRINT activities were designed to be conducted in person. This was infeasible, given the time, distance, and multiple location constraints of project advisors’ participation. As a result, many activities required modifications to approximate the intended function of the original activities. For example, the voting exercise and generating heat maps in Stage 2 were meant to be conducted in person with a group discussion. We made modifications by using the Qualtrics system for creating a Web-based survey paired with a Zoom meeting to enable discussion and screen sharing between each advisor and the lead author. In addition, tutorials needed to be developed and built into the Web-based survey (eg, how to make a vote and practice voting). It was crucial that this data collection tool development go through more than one iteration. We worked with an advisor from the support office to ensure the survey made sense, used sensitive language, and was streamlined enough to reduce potential frustration with completion.

#### Lessons Learned From Conducting Activities

Although Stages 1 to 6 all informed the design process, 1 specific opportunity for further enhancement was at Stage 6. We conducted a hybrid webinar survey to connect during a quarterly dietitian meeting. The concept of the AFINI-T system was completely new to the majority of participants, which made it difficult to build rapport with this group. However, we believe that at this stage of the design process, this was a strength; this may have helped participants provide candid, objective feedback. That said, there were several examples of difficulty in keeping webinar participants engaged. For example, the webinar was run with a brief adjournment for completion of a survey that was then used to encourage group discussion. The ability to take a poll during the webinar may have been more effective at keeping engagement. In addition, the method by which participants attended was inconsistent across locations. For example, most participants joined individually; however, at venues where multiple participants joined from 1 location (eg, RD, director, and assistant director of food services), they filled out the corresponding survey together as well. This may have resulted in bias in some of the feedback collected but also enabled conversation and collaborative thought. Given the exploratory, qualitative nature of the feedback received during this stage, it does not undermine the results of previous stages and, for Stage 6, may have resulted in more critical appraisal from potential group discussion.

### Timeliness in the Time-Constrained Dementia Care Context

One substantial difference between previous work on developing technology for consumer-centered nutrient intake tracking [17-20] and the work presented in this paper is that the purpose of our technology is to support tracking in a regulated LTC environment. This means considerations regarding consumer uptake and use are different than with general consumer market. For example, the novelty does not arise from tracking food and fluid intake per se; this is something that is already mandated for at-risk residents. Instead, the novelty is in improving the method for tracking beyond the current system in place. Other research involving diet tracking apps tends to focus on weight loss and is meant for tracking of an individual’s food intake by the individual. Here, we seek to leverage LTC as an infrastructure already in place to conduct more efficient mandated multiperson monitoring.

The role of nutrition as part of a holistic care plan for individuals living with dementia is discussed in the 2015 European Society for Parenteral and Enteral Nutrition guidelines. They indicate that malnutrition contributes to disease progression and increased caregiver burden and that “nonpharmacological strategies like nutritional interventions are of particular interest as part of disease management” [54]. There is evidence to suggest that adhering to a particular pattern of dietary intake (eg, the Mediterranean diet) is associated with reduced cognitive decline [55]; however, these authors state “more conclusive evidence is needed to reach more targeted and detailed guidelines to prevent or postpone cognitive decline.” Leveraging the necessity to monitor at-risk residents living in LTC through a novel, objective approach to food intake tracking may be beneficial for gaining new insights for defining guidelines.

Specifically considering the dementia care context and nutrition’s role in the process, according to a 2016 systematic review [56], relatively few interventions have been conducted to explore the effect of food intake in mild cognitive impairment or dementia. They conclude that all 43 controlled interventions were at risk of bias and resulted in no consistent evidence either in support or against the effectiveness of nutrition-focused interventions [56]. By providing an alternative method for tracking, we seek to improve upon how these allocated resources are used and aim to provide more informative data. One future direction of the AFINI-T system is to use artificial intelligence to learn food preferences. Circling back to feedback we received in Stage 6, we wish to clarify that through this approach, the AFINI-T system may support caregivers’ efforts in promoting enjoyment of food consumed for residents with communication changes as part of living the dementia journey. Within the scientific community context, in addition, the proposed AFINI-T system may enable knowledge discovery through a thorough automated approach to understanding dietary patterns in the LTC context and beyond.

### Limitations

Between workshop participants and project advisors, 27 unique collaborators representing 15 different roles were engaged in this participatory iterative design process. This sample size is consistent with recent analogous health care–related, user-centered design as well as usability and feasibility studies [57-65], with sample sizes ranging from 5, as in the study by Khan et al [61], to 32, as in the study by Roberts et al [65]. Between 11 and 13 additional participants were involved in the webinar exercise and contributed to 9 survey responses (several individuals filled out a response together). Therefore, the total sample size ranged between 35 and 40; however, not all collaborators contributed to every aspect of the process (eg, user testing in Stage 5 comprised a subsample of 6 individuals). Although this sample size is consistent with early pilot-project prototyping [26,57-65], generalizability remains unclear. As the team of project advisors was relatively small and from the same organization, it will be important for the final product to be tested with a larger sample of users to make sure that the concepts captured more broadly generalize well to users’ needs.

In terms of the physical design requirements, additional discussion is required, as the exact location to house the system remains unclear, as do size restrictions. What was gleaned, however, is that the AFINI-T system must work on the iPad, as this is what is currently in use. The acceptable level of accuracy target was not well defined with project advisors. That said, we can turn to the literature for some insight and important context. There is a tendency for frequent overestimation of food consumption [14,16]; in terms of degree of inaccuracy, estimates of food intake are typically over 50% for food items [16,66], with reported overestimation of food 22% of the time [14]. Furthermore, the source of error is said to be random [66], implying compensation is not possible with current methods. With the AFINI-T system, we should set our targets to be much more stringent, as the automated image-based system removes subjectivity. Careful documentation and exploration of the conditions where the system does not perform optimally will be necessary. One challenging situation is plates where the food items get mixed up over the course of the meal. However, even more crude estimates, where we assume equal eating distributions across types of foods for a plate average, would still improve on the current system as it eliminates subjectivity and reflects relative changes in mass and volume. In terms of time requirements and concerns raised in Stage 6, this is valid and is a next step. When the fully functional prototype is developed, it will be important to evaluate task completion time. Even if the AFINI-T system requires a comparable amount of time, it will yield a trove of powerful nutritional insights so direct comparison of approaches may be more complex than a simple timed trial.

Although it was clear that the project advisors were relatively diverse, no demographic information was collected; this should be considered moving forward, especially when recruiting for a larger sample for user testing. A larger sample size for the final prototype will help deepen our understanding of usability. Finally, given the stage of this research, qualitative analyses were limited to extracting overarching themes across sources; an additional avenue for future work, pending completion of a high-fidelity prototype, is to conduct a more thorough qualitative analysis vetted in an evaluation framework (eg, grounded theory or narrative content analysis) alongside prototype testing and evaluation.

### Conclusions

The purpose of this research was to conduct a multistage participatory iterative design sprint of a Goldilocks quality horizontal prototype for the AFINI-T system. Through input from 38 unique collaborators representing 15 distinct roles, design decisions were informed through the application of this user-centered participatory iterative design sprint. Output from these various stages suggest that although careful consideration for integration with the PointClickCare system is needed, as well as, more generally, policy expectations, project advisors are keen to try a technology like this. Advisors seem to be engaging with the AFINI-T prototype, are receptive to the idea, and are enjoying it. This modified participatory iterative design sprint was effective at understanding the problem space, making informed design decisions, and evaluating receptivity to a novel prototype, all within a compressed period of time (ie, 6 weeks). Next steps for the AFINI-T system include incorporation of learnings from this process and the development of a fully working prototype for additional user testing. We recommend this approach to others for general technology development.

## Appendix

Multimedia Appendix 1 Overview of Stages 2 and 3 including purpose, methods, and results.

Multimedia Appendix 2 Summary of key inspiration concepts from commercially available online healthcare tools. Numbers in brackets correspond to corresponding design decisions.

Multimedia Appendix 3 A summary of the Ravden usability checklist evaluation conducted by 2 technical experts; section 9 was removed as it was not applicable to this version of the prototype.

## Abbreviations

AFINI-T: Automated Food Imaging and Nutrient Intake Tracking
LTC: long-term care
MOHLTC: Ministry of Health and Long-Term Care
PSW: personal support worker
RD: registered dietitian
RIA: Research Institute for Aging
RTLX: Raw Task Load Index
SUS: Subjective Usability Scale
