# Supplementary material for: Prototyping the Automated Food Imaging and Nutrient Intake Tracking System: Modified Participatory Iterative Design Sprint
Source: JMIR Hum Factors. 2019 May 9;6(2):e13017. doi: 10.2196/13017 (PMC6532336; doi:10.2196/13017)
Supplement: Multimedia Appendix 1 [file humanfactors_v6i2e13017_app1.pdf]

Multimedia Appendix 1: Overview of Stages 2 and 3 including purpose, methods, and results.

STAGE 2: Reflect and Storyboard

STAGE 2: Purpose

The purpose of Stage 2 was to use storyboarding to generate solution concepts of the user interface and system output that reflect Stage 1's identified needs and priorities for project advisors' critique in Stage 3.

STAGE 2: Methods

The data from Stage 1 design ideation was combined with the heuristics outlined below to create a series of storyboard solution concepts. Each storyboard was designed using Balsamiq and included tailored concepts developed for three types of primary users identified in Stage 1: a PSW, registered team, and RD. As the system is expected to run on iOS based software and hardware to mesh with the current charting practice on iPads, storyboards were loosely based on iOS Human Interface Guidelines [29]; general iOS expectations will need to be balanced with the current electronic health record system in place (i.e., PointClickCare).

Usability was considered from two perspectives; the designer perspective (Stage 2) and the user perspective (Stages 5 and 6). In Stage 2, we explored usability from the designer's perspective by applying the heuristics outlined by Shneiderman's 8 golden rules [33] and Nielsen and Molich's 10 user interface design heuristics [32] as well as considering heuristics to support trust cues and credibility [60,61] while adhering to best practices for user interface design [28–31]. For example, as shown in Figures 2-3, buttons were designed in accordance with the affordance principle where visual cues act as clues to suggest how an object might be used [28,30] and informative labels [31] were included to reinforce affordances. Colour was used to make the buttons appear actionable [30,31] and users were "rewarded with visual feedback" [31] in the form of confirmation pop-ups, as well as warning and success screens. Finally, inspiration was drawn from three healthcare record systems (e.g., PrognoCIS EHR, ChiroSpring, and Aprima EHR [62–64]) noted as the top electronic medical records software from 2018 online reviews [65,66].

STAGE 2: Results

Design decision inspirations were drawn from PrognoCIS EHR, ChiroSpring, and Aprima EHR [62–64] (see Multimedia Appendix 1 for more detail). These inspirations led to a full, yet organized screen for each of the three interfaces (Figures 2, 3). Many of the inspiration examples and informed design decisions described in Table 2 are illustrated as the output from Stage 2's storyboard critiques (Figures 2, 3). These figures also depict how Stage 2 output informed Stage 3 (including the heatmap overlay in the Stage 2 panes) and Stage 4 (discussed further in subsequent stages).

STAGE 3: Storyboard Critiques

STAGE 3: Purpose

The purpose of Stage 3 was to use the storyboards created in Stage 2 to collaborate with experts to establish design directions and to finalize solution concepts for incorporation into Stage 4's design of the Goldilocks quality prototype.

STAGE 3: Methods

Five participants self-selected as project advisors during Stage 1's workshop from the perspectives of PSW, dining lead, LTC RD, food and nutrition consultant, and food/dietary aide. Similar to the sprint process described by [22], storyboard critiques were conducted with each participant. Feedback was gathered through in-person meetings or over a virtual screen sharing teleconference (a Zoom meeting) when it was infeasible to meet in person on areas of interest, utility, or needing improvement using a wire diagram prototype mockup developed in Stage 2. The first author transcribed feedback in real-time with on-going participant clarification and confirmation. The outputs from Stage 3 included spatial heatmaps on preferred design elements and qualitative feedback for additional consideration. These heatmaps provided feedback similar to the vote with dots exercise described in Stage 1 where more popular concepts received more votes.

STAGE 3: Results

In Figures 2-4, the heatmap overlaid on the Stage 2 pane illustrates the most promising concepts voted by project advisors (e.g., drop-down items and meal-specific tabs). The lower left-hand pane depicts additional considerations captured through discussion. For example, on the PSW interface, building a database of pre-meal images to save time along with the domain knowledge that this solution would work for around 90% of the population (i.e., 10% may require special pre-meal images due to residents receiving non-standard portion sizes).

When design decisions were made, the advisor's perspective was considered and weighed accordingly. As the team of project advisors was relatively small, it will be important for the final product to be tested with a larger sample of users to make sure concepts captured generalize to users' needs more broadly. Generally, the project advisor feedback on storyboard solution concepts (Stage 3) were well received with project advisors actively engaged (e.g., "this system will give me confidence on neighbourhoods I don't know."). Regarding the solution concepts considered together, one advisor said, "This is a fantastic tool for dietitians to use. I can see it is needed. It will advance the profession and advance quality of care for residents. And it will happen in my lifetime". There were no negative comments.
