# Supplementary material for: Prototyping the Automated Food Imaging and Nutrient Intake Tracking System: Modified Participatory Iterative Design Sprint
Source: JMIR Hum Factors. 2019 May 9;6(2):e13017. doi: 10.2196/13017 (PMC6532336; doi:10.2196/13017)
Supplement: Multimedia Appendix 2 [file humanfactors_v6i2e13017_app2.pdf]

Multimedia Appendix 2. Summary of key inspiration concepts from commercially available online healthcare tools. Numbers in brackets correspond to corresponding design decisions; many are highlighted in Figures 2-3.

	Inspiration Example	AFINI-T Prototype Design Decision
**PrognoCIS EHR** [63]		
	Click saving features (1)	Smart tabs opening based on time of day.
	Tap on name (2)	Tap on a name to open the profile for loading a resident profile.
	Clinical snapshot (3)	"Today's Intake Summary" clinical snapshot pane.
	One check default clicks (4)	"select all" capability for registered nursing team referral.
	Input and edit notes (5)	Ability to add and edit notes
**ChiroSpring** [64]		
	Customizability (6)	Planned: panes to be moved, expanded, minimized.
	Solution from scheduling to billing to claims to task management (7)	Supports the process from intake tracking to referrals to further investigation.
	Ability to skip questions (8)	Incomplete data can be entered and edited later or skipped (with a warning).
	Last and current visit notes visible(9)	RD pane (not shown) has "Notes History" right beside "Today's Notes"
**Aprima EHR** [62]		
	Hand off from one person to the next (7)	Supports the process from intake tracking to referrals to further investigation.
	Adaptive learning capabilities with intelligent navigation (10)	Planned: smart food suggestion/selection based on learned preferences and already selected items. Automatically highlighting/changing focus after sub-tasks completed (e.g., progress note pane becomes in-focus after intake completed)
