# Supplementary material for: Prototyping the Automated Food Imaging and Nutrient Intake Tracking System: Modified Participatory Iterative Design Sprint
Source: JMIR Hum Factors. 2019 May 9;6(2):e13017. doi: 10.2196/13017 (PMC6532336; doi:10.2196/13017)
Supplement: Multimedia Appendix 3 [file humanfactors_v6i2e13017_app3.pdf]

Multimedia Appendix 3. A summary of the Ravden usability checklist evaluation conducted by two technical experts; section 9 was removed as it was not applicable to this version of the prototype.

Section	Mode Rating	Expert 1	Expert 1	Expert 2	Expert 2
		% of valid “Always” ratings (n/N)	% of valid “Most of the time” ratings (n/N)	% of valid “Always” ratings (n/N)	% of valid “Most of the time” ratings (n/N)
Section 1: VISUAL CLARITY	Very Satisfactory	73% (11/15)	27% (4/15)	50% (7/14)	50% (7/14)
Section 2: CONSISTENCY	Very Satisfactory	91% (10/11)	5% (1/11)	73% (8/11)	27% (3/11)
Section 3: COMPATIBILITY	Very Satisfactory	79% (11/14)	21% (3/14)	64% (9/14)	36% (5/14)
Section 4: INFORMATIVE FEEDBACK	Very Satisfactory	75% (9/12)	25% (3/12)	69% (9/13)	31% (4/13)
Section 5: EXPLICITNESS	Very Satisfactory	91% (10/11)	9% (1/11)	83% (10/12)	17% (2/12)
Section 6: APPROPRIATE FUNCTIONALITY	Very Satisfactory	100% (8/8)	0% (0/8)	88% (7/8)	13% (1/8)
Section 7: FLEXIBILITY AND CONTROL	Satisfactory/ Very Satisfactory	56% (5/9)	22% (2/9)	89% (8/9)	11% (1/9)
Section 8: ERROR PREVENTION AND CORRECTION	Very Satisfactory	89% (8/9)	11% (1/9)	100% (7/7)	0% (0/7)
Section 10: SYSTEM USABILITY PROBLEMS[a]	No Problems	71% (15/21)	29% (6/21)	81% (17/21)	19% (4/21)

[a] Section 10 was reverse coded. Instead of % of valid “always” and “most of the time”, these columns refer to “% of valid no problems” and “% of valid minor problems”.
